# The Effect of Abamectin on Locusta Migratoria Neurosecretory Cells and Mid Gut, Using Ultrastructure Examination, Oxidative Stress Study, and In-Silico Molecular Docking

**DOI:** 10.3390/molecules28196956

**Published:** 2023-10-06

**Authors:** Nirvina Abdel Raouf Ghazawy, Amira Afify, Ibrahim Taha Radwan, Hanaa Ghabban, Abeer Mousa Alkhaibari, Hattan S. Gattan, Mohammed H. Alruhaili, Abdelfattah Selim, Mona M. Ali Saad

**Affiliations:** 1 Department of Entomology, Faculty of Science, Cairo University, Giza 12613, Egypt; 2 Supplementary General Sciences Department, Faculty of Oral and Dental Medicine, Future University in Egypt, Cairo 11835, Egypt; 3 Department of Biology, Faculty of Science, University of Tabuk, Tabuk 71491, Saudi Arabia; 4 Department of Medical Laboratory Sciences, Faculty of Applied Medical Sciences, King Abdulaziz University, Jeddah 21363, Saudi Arabia; 5 Special Infectious Agents Unit, King Fahad Medical Research Center, King AbdulAziz University, Jeddah 21362, Saudi Arabia; 6 Department of Clinical Microbiology and Immunology, Faculty of Medicine, King AbdulAziz University, Jeddah 21362, Saudi Arabia; 7 Department of Animal Medicine (Infectious Diseases), Faculty of Veterinary Medicine, Benha University, Toukh 13736, Egypt

**Keywords:** *Locusta migratoria*, abmactin, histopathology, enzyme activity, molecular docking

## Abstract

(1) Background: Few studies have been carried out to appraise abamectin toxicity toward *Locusta migratoria* nymphs. (2) Methods: This study aimed to evaluate the cytotoxic effect of abamectin as an insecticide through examining the changes and damage caused by this drug, in both neurosecretory cells and midgut, using *L. migratoria* nymphs as a model of the cytotoxic effect. Histopathological change in the brain was examined in both normal and abamectin-treated fifth-instar nymphs. Neurosecretory cells (NSCs) were also examined where there were loosely disintegrated cells or vacuolated cytoplasm. (3) Results: The results showed distinct histological changes in the gastrointestinal tract of *L. migratoria* nymphs treated with abamectin, with significant cellular damage and disorganization, i.e., characteristic symptoms of cell necrosis, a destroyed epithelium, enlarged cells, and reduced nuclei. The observed biochemical changes included an elevation in all measured oxidative stress parameters compared to untreated controls. The malondialdehyde activities (MDAs) of the treated nymphs had a five- to six-fold increase, with a ten-fold increase in superoxide dismutase (SOD), nine-fold increase in glutathione-S-transferase (GST), and four-fold increase in nitric oxide (NO). (4) Conclusions: To further investigate the theoretical method of action, a molecular docking simulation was performed, examining the possibility that abamectin is an inhibitor of the fatty acid-binding protein Lm-FABP (2FLJ) and that it binds with two successive electrostatic hydrogen bonds.

## 1. Introduction

Insecticides are important in human health and global food production, but there is concern about their potential negative environmental impacts. It is important to identify the minimum required insecticide concentrations for efficacy in pest control, as well as their ecological impacts. One of the most significant and critical pests is the migratory locust, *Locusta migratoria* (Orthoptera, *Acrididae*) [1], which causes widespread and severe crop damage throughout most of Asia and Africa. In many parts of the world, migrating locusts are responsible for significant losses in agriculture during their gregarious phase (moving from a solitary to a group lifestyle) where they cause severe damage to crops and grazing [2,3]. Such impacts result in consequences at the global scale for national economies, societies, and the environment [4,5,6]. For impacted regions of Africa and Asia, an *L. migratoria* invasion has repercussions on food security [7]. Historically, organochlorine pesticides were primarily used to control *L. migratoria,* but these pesticides have been outlawed since the middle of the 1980s due to their chronic toxicity in vertebrates including humans and their persistence in the environment. To combat locusts, insecticides with shorter persistence were developed, such as the organophosphate insecticides fenitrothion and malathion, which were frequently sprayed over huge areas in blanket applications [8,9]. Regular and widespread usage of these pesticides resulted in the development of resistance in locusts [10]. The emergence of insecticide-resistant strains and a desire to reduce the use of synthetic chemicals in the environment has led to alternative control methods being investigated. Interest has grown in using plant extracts that have a low toxicity to mammals and other locust predators, as well as a high rate of bio-degradation in the environment [11,12,13].

**Figure 1:**
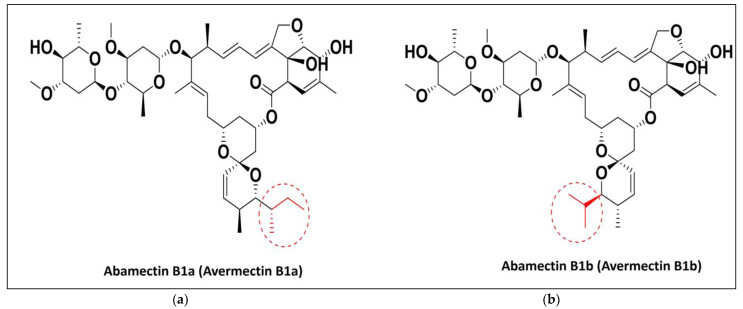
Chemical structure of abamectins (**a**) abamectin B_1a_ and (**b**) abamectin B_1b._

Alkaloids, terpenoids, phenols, flavonoids, steroids, and other allelochemicals produced by the secondary metabolism of plants may provide a safer source of pesticides as a substitution for persistent synthetic insecticides [14,15]. More than 200 plant species have been identified as having insecticidal characteristics, but only a small fraction of these have undergone adequate evaluation [11]. For example, experiments were carried out to determine the impact of a crude methanolic extract from the endemic Tunisian plant *Pergularia tomentosa* on the development and physiology of *L. migratoria* nymphs. *P. tomentosa,* a member of the Asclepiadaceae family, has garnered increased attention due to its antibacterial capabilities [16], molluscicidal activities [17], and insecticidal activity [18]. The soil actinomycete bacterium *Streptomces avermitilis* produces alleochemicals (secondary metabolites) called macrocyclic lactones. Some of these are avermectins, which have insecticidal and anthelmintic properties, one of which is abamectin (ABA) [19,20,21,22,23]. In the laboratory, *S. avermitilis* naturally ferments several types of broth to produce avermectins. Abamectin is one of these, composed of a mixture of two avermectins: avermectin B1a (more than 80%; Figure 1a) and B1b (less than 20%; Figure 1b).

Abamectin, a brand-new insecticide from the avermectin family, was found to be extremely toxic to maize weevils, with an LC_50_ value of 7.26 ppm [24]. It has demonstrated variable toxicity to lepidopterans, and is considered a powerful broad-spectrum acaricide and narrow spectrum insecticide [25]. Abamectin has unmatched and comprehensive antiparasitic action, effective against a wide variety of nematodes, arachnids, and insects [21]. It is employed as the insecticide and nematicide active ingredient for agricultural application, as well as a pest control agent in cattle [26].

Abamectin’s effectiveness against desert locust nymphs was assessed in both lab and field settings. To manage agricultural pests, Egypt uses the insecticide abamectin. A natural byproduct of soil actinomycetes is abamectin [27]. Abamectin consumption appears to be linked to intestinal intoxication and epithelial cell changes, which alter the physiology and function of the pest’s alimentary canal and result in death. The main ingredient in the fermentation that demonstrated the potential to control mites and insect pests on a number of agricultural and horticultural crops around the world is avermectin B1 (abamectin) [28]. Also, abamectin has a great effect on the carp respiratory system, so in this study, it was used to specially target insects, showing its effect on insect tracheae [29].

In addition to promoting the safety of people and the environment, abamectin has demonstrated low toxicity to non-targeted beneficial arthropods, which aids in its inclusion into integrated pest management (IPM) programs [30].

The purpose of this study was to assess abamectin’s efficacy on *L. migratoria* in order to reduce or restrict its harmful damage on the crops.

## 2. Results

### 2.1. Brain Dissection and Ultrastructure Examination

**Figure 2:**
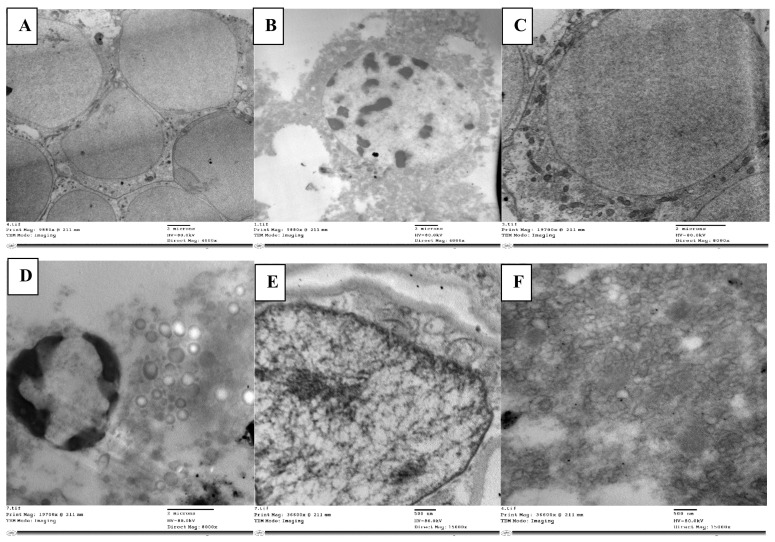
Normal neuroblasts in the brain of *L. migratoria* nymphs are depicted as clear cells without material accumulation in (**A**), while degenerated cells with trapped neurosecretory materials and vacuolated matrix are seen with disorganized axons (**B**). Normal cell with glial cells (gls) surrounding it (**C**). Cells that appeared loose with numerous vacuoles, an accumulation of glycogen (gly), and lysosomal bodies (lys) (**D**). Nucleus with normally dense chromatin and a definite nuclear membrane (**E**). Loose nuclear material that cannot be seen and vacuoles that are plainly visible (**F**). n—nucleus; dch—dense chromatin; nm—nuclear membrane; v—vacuole.

The mortality rate of *L. migratoria* treated with abamectin was 97% at 1000 ppm. In the axons of the control (non-abamectin-treated) neurosecretory cells, there were numerous neurosecretory granules. This is expected and is due to the free movement of neurosecretory granules between the main cell body and the axon. The cell matrix appeared homogeneous, and the neurosecretory cells had clear outlines (Figure 2A).

Conversely, in the neurosecretory cells of abamectin-treated nymphs, the cell matrix appeared heterogeneous (Figure 2B). There were clearly visible neuroblasts and messy granule aggregation, indicating an accumulation and aggregation of neurosecretory materials. There was sufficient trapped material to present a visibly darkened region.

More of the neurosecretory cells had inclusion masses than in the control group, and many vacuoles had developed. The axons appeared empty of granules, likely due to the granule clumping in the main cell matrix, preventing liberation into the axons. The cells also appeared entirely degenerated without regular outline.

Neuroblast cells are normally surrounded by neuropile glia, which fills the interstitial spaces between other neural cells. Neuropile glia are typically rich in microtubules. Kenyon cells are typically scattered around the edge of these cells. In our control cells, this was the structure, with well packed neuropile glia (Figure 2C). However, the neuropile glia in the treated nymphs (Figure 2D) seemed loosely packed with many vacuoles and atypical gaps in the neural tissue, unfilled by glia. Organelles for reunifying separated cells (filopodia) were also present, a characteristic associated with cell division in insect cells. The usual Kenyon cell distribution around the was also lacking.

Abamectin-treated larvae cells had an unusual abundance of lipid droplets, indicating significant cell lysis, and disintegrating neuroblasts were evident. The many vacuoles and lysosomal bodies dispersed throughout the tissue were also indicative of cell lysis. Giant axons were wrapped in the tract glia, and round glycogen granule clusters of various staining intensities were found throughout the glial cytoplasm.

Less chromatin was seen in cells of the abamectin-treated nymphs than in the controls, with no obvious nuclear membrane (Figure 2E). Unlike the disordered cells, vacuoles, lysosomal bodies, and fragmented cell matrix in the treated nymph cells, the chromatin in the control cells showed a uniformly clear nucleus and a clear visible nuclear membrane with uniformly dense and normally distributed chromatin, indicating a homogeneous cell matrix (Figure 2F).

### 2.2. Midgut Dissection and Ultrastructure Examination

**Figure 3:**
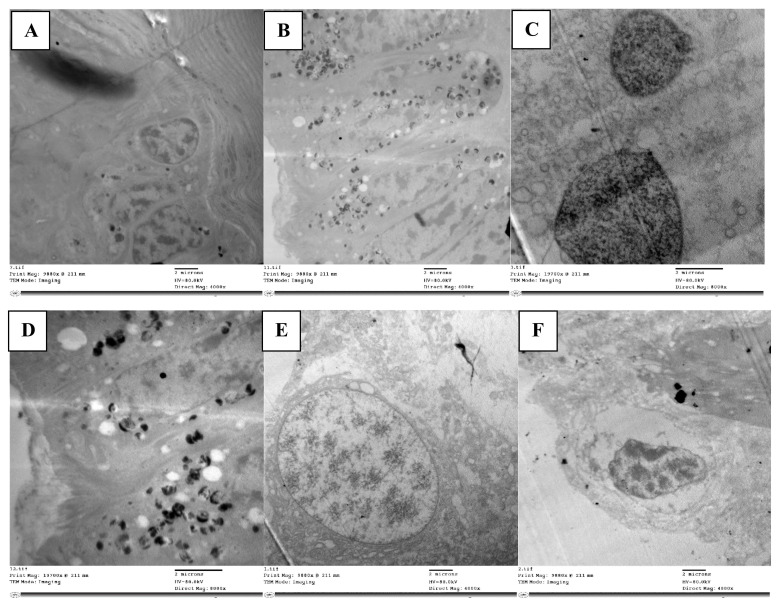
TEM micrograph showing epithelial cells with normal nucleus (n) resting on basement membrane, mitochondria (m) with cristae, and clearly smooth endoplasmic reticulum (ser) (**A**). Damaged and disorganized epithelial cells with vacuolated cytoplasm (v), organelles not detected and many lysosomal bodies (lys) in treated locust (**B**). TEM micrograph in control showing normally chromatinated nucleus (chn) with intact nuclear membrane (nm) and homogenous matrix (**C**). Nucleus disintegrated with less dense chromatin (n) and vacuolated cytoplasm (v) (**D**). The nucleus shrank (n) in treated nymph and appeared detached from nuclear membrane with heterogeneous matrix (M) (**E**). TEM micrograph in control showing normal appearance of nuclear membrane (nm) surrounding nucleus (n) (**F**).

The midgut, the longest and most efficient portion of the alimentary canal, is where digestion and absorption take place. The ultrastructure in the control group midgut epithelium revealed regular cells with a clear proper nucleus (Figure 3A). There was a well-developed muscle layer beneath the basement membrane. The cytoplasm was homogeneous, and the organelles were easily seen and evenly distributed; there were clear mitochondria and a smooth endoplasmic reticulum with cristae. The nucleus was clear, with tightly packed chromatin, and noticeable nucleoli, with an intact nuclear membrane (Figure 3C). There were cristae on the mitochondria. As well as evenly spaced-out organelles, the nucleus had a typical size and rounded shape with an intact nuclear membrane enclosing it (Figure 3F).

The abamectin-treated nymph’s midgut epithelium displayed noticeable differences to the control. The epithelial cell layer appeared disintegrated without septae between cells, large intercellular spaces were present between epithelial cells, and many of the cells had lost contact with each other. The cytoplasm had many vacuoles (Figure 3B) and organelles had disintegrated, presenting an abnormal appearance. Numerous lysosome bodies were distributed in between cells, indicating cell lysis. The cytoplasm was loose, with many autophagic vacuoles and damaged cells, indicating apoptosis (Figure 3D). The nucleus was less densely packed than in the control, although with chromatin and a nuclear membrane visible.

Phagolysosomes were present in the cytoplasm, a marker of ultrastructural changes and cell degradation. In addition to vacuoles in the cytoplasm, the epithelial cell had shrunk unevenly, becoming densely compressed, and with fragmented nucleoli (Figure 3E). Mitochondria deformed to varying degrees due to internal component clumping, membrane rupture, and cristae disintegration. There were also numerous large vacuoles at the margins of the cells.

Insect respiratory systems are composed of tubules that branch out throughout the body to supply oxygen to every part of the body, called tracheae. As they branch through the tissues and cells, the trachea divides into small tracheolethe tubes (tracheoles), which get progressively smaller. An insect trachea has a closely ringed appearance, due to folds or thickenings of the intima. The intima are an ectoderm invagination that protrude on the inner surface of the tracheoles and prevent the tubes from collapsing under pressure. These tiny circular or spiral ridges are called taenidia. The taenidia wind spirally through the membrane wall. Tracheoles convey air from outside the insect deep into the body, with the branches terminating in minuscule blind ends, where aerobic gas exchange occurs. Hemolymph (analogous to blood in mammals) is present at these locations and transports oxygen to muscles and nerves.

**Figure 4:**
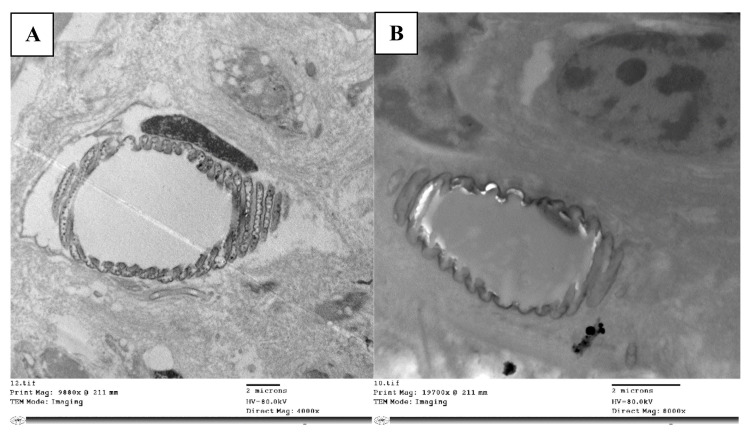
TEM micrograph the trachea is dividing into taenidia and a small tracheole (**A**), while the treated nymphs’ tracheae lacked extracellular vesicles (resilin particles) (**B**).

Within tracheoles, there are extra-cellular vesicles (Figure 4A) that have membrane-like cell structures that are thought to be essential for intercellular communication. In the tracheole lumen of the control nymphs, these vesicles were present, but in the abamectin-treated nymphs, the extracellular vesicles (resilin particles) were absent (Figure 4B). Also, the strengthening taenidia were irregularly shaped and discontinuous.

**Figure 5:**
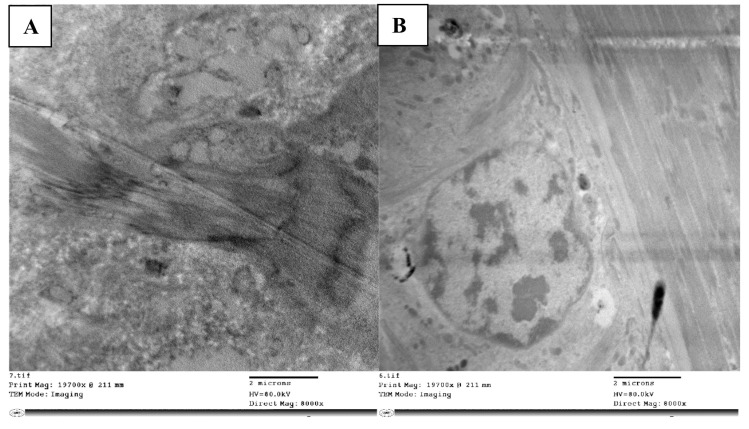
TEM micrograph showing normal muscles (m) with dark (d) and light bands (l) and clear Z-disc (z) (**A**), while muscles appeared without normal striations (m) and loosely detached with unobvious Z-disc and cracks appearing in between (cr) (**B**).

In a normal nymph, the epidermal cell layer is followed by a separate muscle layer. A typical cytologically striated structure and pronounced Z lines can be seen in this muscle layer (Figure 5A). It helps with both contraction and relaxation during molting. Muscle fiber myofibrils are organized radially, with mitochondria and peripheral nuclei separating them from one another. Myofibrils have alternating bright and dark bands of filaments (I and A bands) which contain actin and myosin, respectively. Within the I band, there is a dark center membrane called the Z line, which is the contractile part (sarcomere). Given that it is substantially denser in contractile filaments, this line can be accurately characterized as a form of continuous transverse septum.

With abamectin-treated nymphs, the muscle fibers were vacuolated, disorganized, and damaged and the bands and zones were far less distinct than with the control. The characteristic striations that typically divide light and dark bands were instead loose vacuolated fibrils with fractures and spaces between them (Figure 5B).

**Figure 6:**
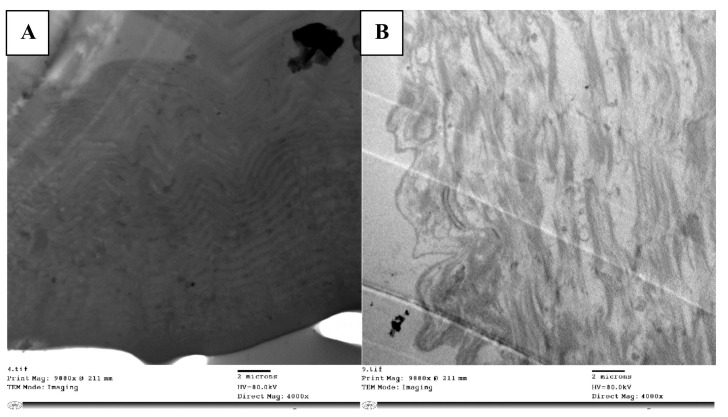
TEM micrograph showing normal cuticle appearing with exocuticle (ex), endocuticle (en), and epicuticle (ep) in homogenous appearance and arrangement layers (**A**), while layers appeared in loose manner, disorganized with intercellular spaces (ics) in between without any arrangement to the layers (**B**).

Control nymph outer cuticles showed normal structure, separated into an outside epicuticle, an exocuticle, and an internal endocuticle. The cuticle lamellae, which are composed of chitin microfibrils stacked horizontally and at a specific angle to one another, are part of the endocuticle’s separate consecutive sheets or lamellae (Figure 6A). The endocuticle lamellae are divided by several vertical columns of oriented microfibrils, often known as pore canal (PC) fibers.

In the nymphs treated with abamectin, the cuticle had separated from the hypodermis, and the endocuticle and exocuticle were indistinguishable. The cuticle’s ultrastructure showed vacuolization, particularly at the exocuticle, between the cuticular lamellae, and at the endocuticle (Figure 6B). The PC fibers were slightly distorted, and the cuticular lamellae’s borders had disappeared in certain locations. There were also numerous cytoplasmic vesicles that are obviously autophagic vacuoles.

### 2.3. Alteration in Oxidative Enzymes of Treated L. migratoria Treated with Abamectin

*L. migratoria* nympths treated with abamectin showed changes in oxidative stress enzymes within the cells, with increases in superoxide dismutase (SOD), glutathione-s-transferase (GST), malondialdehyde (MDA), and nitrous oxide (NO) levels. The concentration of MDA was investigated as a lipid peroxidation biomarker in the hemolymph and fat tissue. As compared to the control, the group treated with abamectin showed a significant six-fold elevation in MDA level in hemolymph and significant five-fold increase in fat tissue, respectively (Table 1). In the hemolymph of the treated group, the activity of SOD was elevated about 10-fold (significant) compared to the controls. The fat tissue of abamectin-treated group also showed marked elevations in the activity of SOD by about eight-fold relative to the controls (Table 1). Hemolymph and fat tissue showed nine-fold elevations in the activity of GST in the treated nymphs relative to the controls (Table 1).

Levels of NO were measured as a pro-inflammatory marker. In the hemolymph and fat tissue, the NO levels increased four-fold relative to the controls. However, as compared to the Abamectin group, the results are shown in Table 1.

### 2.4. Molecular Docking Study

**Figure 7:**
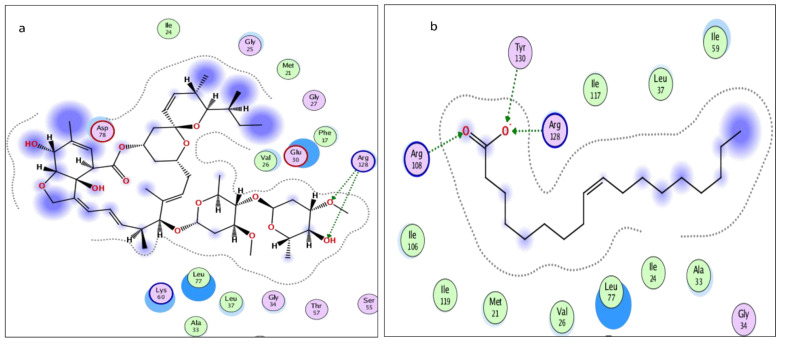
Two-dimensional interactions of (**a**) abamectin and (**b**) OLA (co-crystalized ligand).

**Figure 8:**
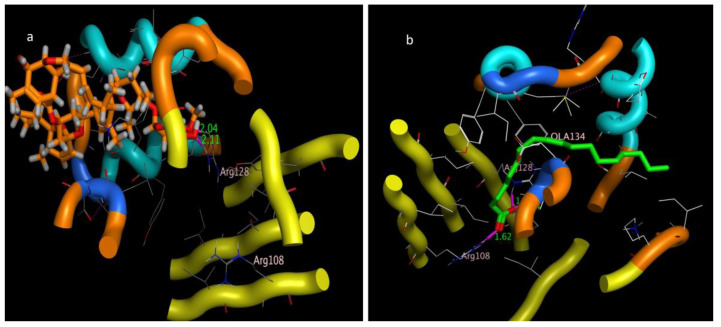
Three-dimensional interaction bond length of (**a**) abamectin and (**b**) OLA (co-crystalized ligand).

**Figure 9:**
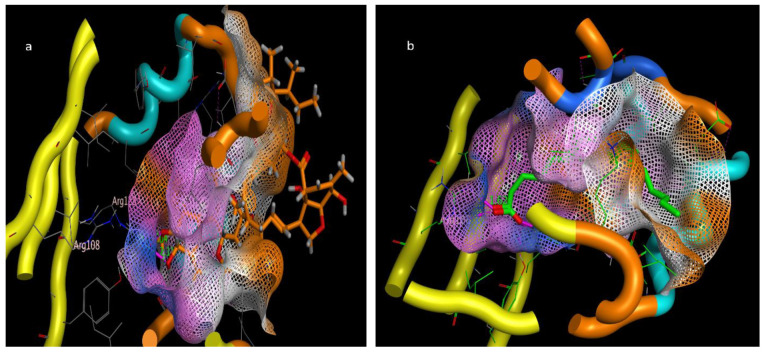
Three-dimensional interactions pose inside pocket of 2FLJ (**a**) abamectin and (**b**) OLA (co-crystalized ligand).

The molecular docking presented in Table 2 revealed that abamectin b could bind to the *Lm*-FABP active site pocket with two hydrogen bonds, with bond lengths of 2.4 and 2.11 Å between the residue with the hydroxyl group and Arg128 with the methoxy group, as shown in Figure 7 and Figure 8. The co-crystallized ligand (control) showed three hydrogen bonds with bond lengths of 1.7, 174, and 1.62 between the residues Try130, Arg128, and Arg108, respectively. The molecular docking study demonstrated that abamectin could bind to the specific protein with successful binding modes and the three-dimensional structure of abamectin inside the active site pocket shown in Figure 9.

## 3. Discussion

This study is one of very few studies showing the impact of abamectin on the neurosecretory cells of the last instar of *L. migratoria*. The significant role played by the neurosecretory cells in the brain during molting and metamorphosis is well known. In the few studies carried out on the effects of abamectin on locust brain tissue and neurosecretory cells, there was evidence of serious cell damage and significant accumulation of neurosecretions, which were not freely released to normal channels through axons [31].

Abamectin, a naturally occurring product of *Streptomces avermitilis* in soil habitats, is commonly employed as an insecticide [32]. Cook et al. [33] state that its mechanism of action is binding to glutamate-gated chloride channels present in nerve and muscle cells, which impairs and disrupts brain development.

To learn more about the toxicity of abamectin, this study examined histopathological changes in the brain of both normal (control) and abamectin-treated fifth-instar nymphs. Abamectin-induced changes were specifically noted in neurosecretory cells, producing loose, disintegrated cells and vacuolated cytoplasm with an accumulation of neurosecretory crystals, whereas in controls, there were lightly stained nuclei and condensed chromatin. These findings concord with those of Miladi et al. [9], who tested *Pergularia tomentosa* extract treatment on locusts.

Abdel Rahman [34] confirmed that abamectin affects the brain and compound eyes of locusts with significant decreases in brain cell numbers, and the protocerebrum, optic lobe, deutocerebrum, and tritocerebrum of the brain all being deformed and shrunken in treated embryos. In insects treated with abamectin, the brain’s neurosecretory system showed a buildup of stainable material in the corpora cardiaca and neuropilar storage areas, which was linked to an absence of ovarian development [35]. The slow rate of these neurosecretory proteins’ transit to the neurosecretory reservoirs was suggested as the cause of their buildup.

Rembold et al. [36] found that azadirachtin disrupts the production of ecdysone in the neuroendocrine system of *L. migratoria*, resulting in a significant buildup of neurosecretory material in the corpus cardiacum. Guiqiang et al. found a considerable reduction in the number of cerebral neurosecretory cells in *L. migratoria* after azadirachtin treatment, with amplified and aberrant axons severed from the cell body [37]. The neurosecretory cells may also burst as a result of azadirachtin treatment due to the accumulation of neurosecretory materials, as seen in *Heteracris littoralis* nymphs [38]. Subrahmanyam et al. [39] hypothesized that azadirachtin interfered with the neuroendocrine system controlling ecdysone and juvenile hormone (JH) syntheses in *L. migratoria* nymphs. They discovered a significant buildup of stainable neurosecretory material and a slow transport rate of neurosecretory proteins in the corpus cardiacum. They also saw that nymphs treated with the insect growth regulator cascade showed a notable decrease in the number of cerebral NC as well as the number of neurosecretory particles carried by the axoplasm. Ghazawy [40] also found that treatment of fifth-instar *Schistocerca gregaria* with lufernuron, a chitin synthesis inhibitor, reduced the release of neurosecretory material from the brain NC in the pars intracerebral.

Our findings of cell damage, and the appearance of vacuoles and lysosomal bodies due to cell lysis, were consistent with the effect of ozone exposure on brain tissues, neurosecretory cells, muscles, and fat body in caterpillars and grubs reported by Ghazawy et al. [41] A corresponding accumulation of neurosecretory granules inside cells, without release into the axons, also occurred in following farnesol treatment in a study by Awad and Ghazawy [42].

We found distinct histological changes in the gastrointestinal tract of *L. migratoria* nymphs treated with abamectin, resulting in significant cellular disorganization and severe damage, as well as the characteristic symptoms of cell necrosis.

The effects of different plant extracts on the digestive system of locusts have previously been compared. Abdellaoui et al. showed that gibberellic acid (GA3), a plant growth regulator, was shown to be the cause of a cytotoxic effect on the digestive system of *L. migratoria* nymphs according to Abdellaoui et al. [43]. The foregut and gastric caeca’s epithelial cells were destroyed by GA3, and the muscle layer covering these organs was torn apart. Similarly, Ammar and N’cir [44] found that desert locust nymphs fed an artificial diet containing 2% Cestrum parquii (Solanaceae) leaf powder had significantly less muscle layer thickness and epithelial cell height in the midgut. *C. parquii* also eradicated the midgut’s extracellular microorganisms.

Our observations using electron microscopy showed that cells exposed to abamectin underwent a variety of changes. In the fat body, ultrastructural changes correspond to changed fat droplets, cell loss, altered nuclei, and the vacuolization of both cytoplasm and mitochondria. These alterations suggest that abamectin exposure affects a variety of organs, tissues, and cells. Sumida et al. [45] found similar ultrastructural alterations in ants treated with boric acid.

We found in our abamectin-treated nymphs that the nucleus was ruptured and faint, and that the mitochondria were fragmented, loose, and lacked cristae. Similarly, Afify et al. [46] found that in *C. pipiens* larvae treated with Cu-chl, the gut cells were disorganized and out of shape, with numerous vacuoles and tissue damage, and that the epithelium had detached from the gut wall, in addition to the midgut’s muscular layers being atrophied. Similar muscle injury was observed in *C. capitata* treated with Phloxine B [47] and *C. pipiens* treated with Cu-chl and Mg-chl in both species [46].

In our study we found that abamectin-treated nymph midgut cells produced cytoplasmic vacuoles. This is a normal response to cytotoxins, with the vacuoles being used in an attempt to store the toxins and prevent them from interfering with cellular metabolism. This effect on locusts was also observed by Pilat [48], who determined that vacuole formation was the first stage of cellular disintegration from both toxins and disease.

The observed collapse of the midgut epithelial cell layer brought on by abamectin in our experiments has also been seen in *Aedes aegypti* treated with a new meso-substituted cationic porphyrin [49] and the naturally occurring substance pellitorine [50]. Other changes we observed in the midgut included cell loss in the epithelium, enlarged cells, and reduced nuclei. As with similar experiments carried out on Sarcophagidea (flesh fly) larvae by Ali et al. [51], the organelles of gut cells also seemed loose, the mitochondria lacked cristae, and the cytoplasm was vacuolated. Organelles were also ruptured with numerous spaces between them, forming the appearance of an epithelium with detached cells and numerous vacuoles, as well as dispersed secretory vesicles. Ali et al. also found that the peritrophic membrane had vanished, and when they treated gut cells with silver nanoparticles, the muscles folded and vacuolated, and loose fatty tissues were seen.

To the best of our understanding, abamectin affects an insect’s antioxidant system. Hence, the current study determined the oxidative stress in *L. migratoria*. Our study revealed a considerable increase in NO, SOD, GST, and MDA activity starting from the first day following abamectin treatment, with slight and insignificant changes from the controls in both hemolymph and fat tissue, which was consistent with El Sayed et al. [52].

The results of this study suggest lipid peroxidation occurred and show that the examined biomarkers were affected by an improvement in the insect antioxidant system’s capacity to scavenge ROS caused by the oxidative stress of abamectin.

Intracellular binding proteins are a family of low-molecular-weight single-chain polypeptides. The major functions of these proteins are to carry lipids and poorly soluble components like fatty acids, retinoids, bile acids, or any other hydrophobic constituent to be transported through the cytosol to different cellular compartments for different purposes of utilization or storage, e.g., fatty acid-binding proteins (FABPs) and their utility in membrane phospholipid synthesis, lipid metabolism, and mitochondrial beta oxidation. A huge number of functional roles of FABPs have been proposed based on physiological disorders in the animal kingdom [53]. Usually, migratory birds and insects contain high levels of fatty acid-binding proteins (FABPs); e.g., in the flight muscle of desert locust (*Schistocerca gregaria*), 18% of the protein contents are FABPs (*Sg*-FABP) [54]. *Locusta moratoria* carries a homologous protein, *Lm*-FABP (4FLJ), which is different from Sg-FABP only in the positions of three amino acids [55]. Based on the molecular docking prediction listed in Table 2, the tested compounds have multiple stable binding modes with the target fatty acid-binding protein (2FLJ). The simulation results confirmed that abamectin could form two strong electrostatic interactions: hydrogen bonding from a hydroxyl and a methoxy group on the abamectin to Arg128 amino acid residues on the target protein. Taking the binding amino acid and bond length of the positive control (inhibitor), OLA, into consideration, the positive control binds to Arg128 the same as abamectin, in addition to two other residues of Try130 and Arg108. The bond distances between the protein receptor and abamectin are 2.04 and 2.11 Å. Such bond length is described to be relatively strong if compared to the bond length made by the control, OLA, which is in the range of 1.62 to 1.74 Å. Additionally, the root mean square deviation (RMSD), which extensively measures the ability of replacing the co-crystalized drug OLA with abamectin, was fairly accepted (RMSD = 1.63 Å) with a high scoring energy (stability-favored pose) of −35.088 kcal/mol. Based on the simulation results of RMSD and the highly negative energy that abamectin achieved, the minimum requirements to replace OLA were met even though the abamectin showed the lowest number of two interactions, with a bond length longer than that made by OLA, which makes the binding affinity of OLA more faveored than abamectin, but the dipole–dipole interactions in addition to pi–pi staking showed by avermectin introduce a convenient reason to be seen as an FABP inhibitor. For good superimposition, the value of RMSD should not exceed 1.7 Å, with low (negatively charged) scoring energy being preferable [56]. Based on our simulation model, abamectin can thus theoretically occupy the active site pocket of *Lm*-FABP in *L. moratoria* and can thus explain its inhibition effects, given a suitable dose.

## 4. Materials and Methods

### 4.1. Rearing of Insect Colony

The locust, *L. migratoria*, was grown at room temperature in a laboratory environment at Cairo University’s Entomology Department at the Faculty of Science. It was fed daily meals of maize in the summer and clover in the winter. Fifth nymphal instars of *L. migratoria* were used in the tests.

### 4.2. Bioassay of Lethal Effects of Abamectin

An abamectin bioassay was performed using a serial concentration with five abamectin dose levels of 0.01, 0.1, 0.5, 1, and 1.5 ppm. These were each prepared in 30 mL of aqueous solution, topping up abamectin with distilled water to produce the desired concentration. For complete solubility, the abamectin solution was sonicated for 15min using a probe Q55 (Qsonica) sonicator.

A contact method was used in this bioassay: 100 g of fresh *Sesbania sesban* leaves was sprayed with 10mL of abamectin, replicating this for each of the five abamectin concentrations. Each was transferred to a 500 mL glass jar, and ten nymphs were placed in each jar. A control jar was the same, with 100 g of *S. sesban* leaves and ten nymphs, but the leaves had been sprayed with 10 mL of water. The glass jars were covered with a fine mesh cloth. Mortality was recorded after 24 h and corrected according to Abbott’s formula [57]. The LC_50_ value and 95% confidence intervals were calculated from probit regressions using the polo-PC computer program [58], with an estimated LC_50_ of abamectin at 58.4 ± 2.11 ppm.

### 4.3. Ultrastructural Examinations

Twenty nymphs (5th instar) were given an abamectin injection and left for 24 h. These treated nymphs and untreated control nymphs had their brains and midguts removed, and this tissue was then pre-fixed in 1% osmium tetroxide for 1.5 h at room temperature, before being fixed in 2.5% glutaraldehyde for 1.5 h at 4 °C. Following tissue dehydration in a series of increasing alcohol concentrations (50, 70, 90, 95, and 100% for 15 min for each concentration), the tissue was implanted in Epon 812 embedding resin. An ultramicrotome was used to cut ultra-thin slices. Transmission electron microscopy (TEM) was performed at the Electron Microscope Unit, Faculty of Agriculture, Cairo University. After sample fixation, the slices were stained with uranyl acetate and lead citrate and analyzed using an electron microscope (JOEL JEM 1400, 80 kvolt) at different magnifications. The images were captured using an optronics AMT CCD camera with 1632-pixel format and slide mount configuration. This camera uses a 1394 fire wire board for acquisition.

### 4.4. Bioassay of Oxidative Stress Enzyme

#### 4.4.1. Initial Tissue Sample Preparation for Enzyme Assays

For each assay described below, 5 g of *L. migratoria* tissue was homogenized in 5 mL of 0 °C phosphate buffer. This was centrifuged at 20,000 rpm for 10 min at 4 °C, and the supernatant was used in the assays below to estimate enzyme activities. The assays were performed in the Cairo University Research Park (CURP), Faculty of Agriculture, Cairo University, using the Sunostk733plus SBA-733PLUS spectrophotometer. The enzyme activity was measured in the same tissue sample in comparison with the control.

#### 4.4.2. Superoxide Dismutase (SOD) Assay

The SOD activity was assessed according to Misra and Fridovich [59], measured as superoxide dismutase (OD) produced per mg protein per minute. The principle of the assay is based on the ability of the enzyme to inhibit the phenazine methosulphate-mediated reduction of nitro-blue tetrazolium salt. After sample lysate (or homogenate) preparation, the chemical reagent quantities were added as recommended, and the absorbance was measured using the spectrophotometer at 560 nm. The percent inhibition was calculated using the following equation:

$$\mathrm{Percent}\;\mathrm{of}\;\mathrm{inhibition}=\frac{A_{cont}-A_{sample}}{A_{cont}}\times 100$$

where *A_cont_* is the control absorbance, and *A_sample_* is the absorbance of the sample.

#### 4.4.3. Glutathione S–Transferase (GST) Assay

The GST activity was determined according to Habig et al. [60] and expressed as OD/Mg protein/min. The total GST activity, caused by the conjugation between CDNB with reduced glutathione, was measured at 340 nm using the spectrophotometer. The rate of increased absorbance due to the conjugation is directly proportional to the GST in the sample according to:

$$U=A_{sample}\times \frac{2.812}{g\;of\;tissue\;used}$$

The GST activity was determined according to Habig, Pabst, Fleischner, Gatmaitan, Arias, and Jakoby [60] and expressed as OD/Mg protein /min.

#### 4.4.4. Malondialdehyde (MDA) Contents

The MDA concentration in extracted tissue samples was measured as described by Jain and Levine [61]. MDA undergoes a chemical reaction with thiobarbituric acid (TBA), forming a colored mixture, and is an indicator of lipid peroxidation. It is calculated as OD/Mg protein/min according to the following equation:

$$U=\frac{A_{sample}}{A_{standard}}\times \frac{10}{g\;of\;tissue\;used}$$

### 4.5. Molecular Docking Study

#### 4.5.1. Molecular Docking of Acetylcholine Esterase Enzyme

1. **Source of the objective protein**

We investigated the binding capability of abamectin with fatty acid-binding proteins from the flight muscle of *L. migratoria* on binding site *Lm*-FABP (4FLJ). This was performed theoretically, using a molecular docking artificial intelligence program, to determine whether the abamectin drug could interact and fix itself inside the binding site. The three-dimensional structure of fatty acid-binding protein 2FLJ was downloaded from the official website of the Protein Data Bank (PDB Code: 2FLJ) (https://www.rcsb.org/structure/2FLJ). The protein was downloaded as PDB format, without post-download alterations. The co-crystallized ligand OLA (oleic acid) was labelled green.

2. **Energy minimization**

Of the two isomers of abamectin, avermectin B1a (isomer B in Figure 1), which represents at least 80% of the abamectin, was chosen as the major content to fulfill the in silico study and to inspect the probability of a bond between its structure and the 2FLJ binding site. The structure of abamectin B was drawn using CAMBRIDGESOFT CHEMOFFICE 2015 Professional 15.0.0 software and saved in the Mol format, followed by selecting the most stable pose via the energy minimization selector anticipating the default option of Amber12: EHT force field function till gradient convergence of 0.01 kcal/mol by Molecular Operating Environment MOE_2015.10. MOE software was installed on a 64-bit operating system with an Intel (R) Core (TM) i5-2400 CPU @ 2.40 GHz and 8 GB RAM system.

#### 4.5.2. Docking Procedure

The reference drug, oleic acid (OLA), and abamectin B were labeled in green and orange colors to be visually distinguished. The binding sites were recognized automatically from the surfaces and maps option, to recall the co-crystallized ligand binding site directly, and the docking was accomplished after ligand and protein justification, anticipating the default sets of the “Rotate Bonds” choice, to permit flexible ligand–rigid receptor docking. The scoring function was modified to be the London G with triangle matcher replacement. Ten conformers were replaced instead of the automatic option of thirty conformers of the best-scoring ligand, and low energetics were retained. The top five conformer scoring of ligand–receptor docking was then shown via two- and three-dimensional ligand–receptor interactions [62].

### 4.6. Statistical Analysis

A statistical software package (IBM-SPSS ver.23) was used to do statistical analyses. The Shapiro–Wilk test illustrated that data were normally distributed. One-way analysis of variance (ANOVA) was used to investigate the effect of treatment on the studied variables. Data are presented as mean ± standard error. The least significant differences (LSD) test was used to clarify the statistical differences among the experimental groups, with *p* < 0.05 considered a statistically significant result.

## 5. Conclusions

Abamectin treatment resulted in changes in the ultrastructure of the neurosecretory cells in the brain, midgut epithelium, muscles, and cuticle in the *L. migratoria* nymphs used in this study. Abamectin can therefore cause cytotoxicity, which controls cell death or apoptosis. A simulated molecular docking study was carried out, using one the most important proteins that coordinate lipid trafficking and signaling in cells, Lm-FABP (2FLJ). The docking simulation revealed that the abamectin could theoretically produce an inhibitory effect through the successful binding of abamectin with the Lm-FABP protein. The predicted (simulated) FABP-inhibitory effect of abamectin may be responsible for the biological paradox regarding the secretion of self-defense enzymes. Our team believe further practical research would help us better understand the cytotoxicity mechanism of abamectin. Studies are also encouraged to better understand the dynamics between oxidative stress induced by abamectin and other environmentally friendly insecticides against pests that have strong antioxidant capability.

## Figures and Tables

**Table 1:** The effect of treatment with Abamectin on enzyme activities. Data are displayed as mean ± standard error.

Enzyme	Tissue	Control	Abamectin	Effect of Treatment
MDA (nmol/mL)	Hemolymph	7.15 ± 0.44	42.10 ± 0.34 *	F_2,12_ = 876.671, *p* = 0.0001 ^#^
	∆	---	5.88	
	Fat	8.68 ± 0.22	44.37 ± 0.73 *	F_2,12_ = 1807.39, *p* = 0.0001 ^#^
	∆	---	5.11	
SOD (U/mL)	Hemolymph	9.00 ± 0.31	86.65 ± 0.43 *	F_2,12_ = 8710.27, *p* = 0.0001 ^#^
	∆	---	9.63	
	Fat	11.14 ± 0.33	90.81 ± 1.07 *	F_2,12_ = 3164.62, *p* = 0.0001 ^#^
	∆	---	8.15	
	Hemolymph	18.72 ± 0.68	163.23 ± 1.60 *	F_2,12_ = 4483.205, *p* = 0.0001 ^#^
GST (U/L)	∆	---	8.72	
	Fat	22.77 ± 1.84	192.92 ± 0.85 *	F_2,12_ = 2942.222, *p* = 0.0001 ^#^
	∆	---	8.47	
	Hemolymph	13.06 ± 0.56	52.49 ± 0.45 *	F_2,12_ = 1375.467, *p* = 0.0001 ^#^
NO (µmol/L)	∆	---	4.02	
	Fat	13.13 ± 0.95	55.04 ± 0.32 *	F_2,12_ = 1346.17, *p* = 0.0001 ^#^
	∆	---	4.19	

*p* < 0.0001 represents significant effect. *: significant difference (*p* < 0.05), as compared to the corresponding controls. #: significant difference (*p* < 0.05), as compared to the corresponding Abamectin-treated group. ∆: fold change in relation to the controls.

**Table 2:** Docking results of oleic acid and abamectin as inhibitor prediction in the vicinity of *Lm*-FABP (2FLJ).

Compound	Interactions	Type	Distance (Å)	Score (kcal/mol)	RMSD (Å)
**OLA**	Tyr130-O	Hydrogen bond	1.74	--	--
	Arg128-O	Hydrogen bond	1.7		
	Arg108-C=O	Hydrogen bond	1.62		
**Abamectin B**	Arg128-OH	Hydrogen bond	2.04	-35.088	1.63
	Arg128-Ome	Hydrogen bond	2.11		

## Data Availability

This article contains all of the data that were created or analyzed throughout the investigation.
